# Benefits of Combining Circulating Tumor DNA With Tissue and Longitudinal Circulating Tumor DNA Genotyping in Advanced Solid Tumors: SCRUM-Japan MONSTAR-SCREEN-1 Study

**DOI:** 10.1200/PO.24.00283

**Published:** 2025-04-10

**Authors:** Takao Fujisawa, Yoshiaki Nakamura, Hideaki Bando, Chigusa Morizane, Masafumi Ikeda, Norio Nonomura, Nobuaki Matsubara, Hiroji Iwata, Yoichi Naito, Susumu Okano, Daisuke Aoki, Kenichi Harano, Naoya Yamazaki, Kenjiro Namikawa, Makoto Ueno, Shigenori Kadowaki, Eiji Oki, Ken Kato, Yoshito Komatsu, Taroh Satoh, Taito Esaki, Tadamichi Denda, Tetsuya Hamaguchi, Kentaro Yamazaki, Nobuhisa Matsuhashi, Hisateru Yasui, Hironaga Satake, Tomohiro Nishina, Naoki Takahashi, Masahiro Goto, Yu Sunakawa, Takeshi Kato, Tomoyuki Otsuka, Hikaru Abutani, Hanna Tukachinsky, Jessica K. Lee, Geoffrey R. Oxnard, Naomi Kuramoto, Satoshi Horasawa, Yasutoshi Sakamoto, Hiroya Taniguchi, Takayuki Yoshino

**Affiliations:** ^1^ Translational Research Support Office, National Cancer Center Hospital East, Kashiwa, Japan; ^2^ Department of Head and Neck Medical Oncology, National Cancer Center Hospital East, Kashiwa, Japan; ^3^ Course of Advanced Clinical Research of Cancer, Juntendo University Graduate School of Medicine, Tokyo, Japan; ^4^ Department of Gastroenterology and Gastrointestinal Oncology, National Cancer Center Hospital East, Kashiwa, Japan; ^5^ International Research Promotion Office, National Cancer Center Hospital East, Kashiwa, Japan; ^6^ Department of Hepatobiliary and Pancreatic Oncology, National Cancer Center Hospital, Tokyo, Japan; ^7^ Department of Hepatobiliary and Pancreatic Oncology, National Cancer Center Hospital East, Kashiwa, Japan; ^8^ Department of Urology, Osaka University Graduate School of Medicine, Suita, Japan; ^9^ Department of Medical Oncology, National Cancer Center Hospital East, Chiba, Japan; ^10^ Department of Breast Oncology, Aichi Cancer Center Hospital, Nagoya, Japan; ^11^ Department of General Internal Medicine, National Cancer Center Hospital East, Chiba, Japan; ^12^ Department of Experimental Therapeutics, National Cancer Center Hospital East, Chiba, Japan; ^13^ Department of Obstetrics and Gynecology, Keio University School of Medicine, Tokyo, Japan; ^14^ Department of Dermatologic Oncology, National Cancer Center Hospital, Tokyo, Japan; ^15^ Department of Gastroenterology, Kanagawa Cancer Center, Yokohama, Japan; ^16^ Department of Clinical Oncology, Aichi Cancer Center Hospital, Nagoya, Japan; ^17^ Department of Surgery and Science, Kyushu University, Fukuoka, Japan; ^18^ Department of Head and Neck, Esophageal Oncology, National Cancer Center Hospital, Tokyo, Japan; ^19^ Department of Cancer Center, Hokkaido University Hospital, Sapporo, Japan; ^20^ Center for Cancer Genomics and Precision Medicine, Osaka University Hospital, Suita, Japan; ^21^ Department of Gastrointestinal and Medical Oncology, National Hospital Organization Kyushu Cancer Center, Fukuoka, Japan; ^22^ Division of Gastroenterology, Chiba Cancer Center, Chiba, Japan; ^23^ Department of Gastroenterological Oncology, Saitama Medical University International Medical Center, Hidaka, Japan; ^24^ Division of Gastrointestinal Oncology, Shizuoka Cancer Center, Shunto-gun, Japan; ^25^ Department of Gastroenterological Surgery and Pediatric Surgery, Center for One Medicine Innovative Translational Research, Gifu University Graduate School of Medicine, Gifu, Japan; ^26^ Department of Medical Oncology, Kobe City Medical Center General Hospital, Kobe, Japan; ^27^ Cancer Center, Kansai Medical University Hospital, Hirakata, Japan; ^28^ Department of Medical Oncology, Kochi Medical School, Kochi, Japan; ^29^ Department of Gastrointestinal Medical Oncology, National Hospital Organization Shikoku Cancer Center, Matsuyama, Japan; ^30^ Department of Gastroenterology, Saitama Cancer Center, Kitaadachi-gun, Japan; ^31^ Cancer Chemotherapy Center, Osaka Medical and Pharmaceutical University Hospital, Takatsuki, Japan; ^32^ Department of Clinical Oncology, St Marianna University School of Medicine, Kawasaki, Japan; ^33^ Department of Surgery, National Hospital Organization Osaka National Hospital, Osaka, Japan; ^34^ Department of Medical Oncology, Osaka International Cancer Institute, Osaka, Japan; ^35^ Chugai Pharmaceutical Co, Ltd, Tokyo, Japan; ^36^ Foundation Medicine, Boston, MA; ^37^ Department of the Promotion of Drug and Diagnostic Development, National Cancer Center Hospital East, Kashiwa, Japan

## Abstract

**PURPOSE:**

The utility of capturing heterogeneity by circulating tumor DNA (ctDNA) genotyping combined with tissue analysis or applying it in a sequential manner remains uncertain.

**METHODS:**

We assessed the clinical value of ctDNA genotyping using data from 2,187 patients with advanced solid tumors enrolled in SCRUM-Japan MONSTAR-SCREEN-1, a nationwide cancer genome screening project, which examined ctDNA from longitudinally collected blood samples and tumor tissue samples (UMIN 000036749).

**RESULTS:**

Among 667 patients with both baseline ctDNA and tissue genotyping results, 51 (7.6%) had actionable biomarkers identified exclusively through ctDNA genotyping. The most frequent targets of genotype-matched therapy guided by solely ctDNA were immune checkpoint, estrogen receptor, and poly(ADP-ribose) polymerase (PARP). Comparison of objective response rates (ORRs) and progression-free survival (PFS) between patients treated based on tissue versus ctDNA alone showed no significant difference, with ORRs of 34.0% versus 23.1% (*P* = .54) and a median PFS of 11.5 versus 13.8 months (hazard ratio [HR], 1.4 [95% CI, 0.72 to 2.80]), respectively. Among 924 patients undergoing sequential ctDNA genotyping, the detection of actionable biomarkers increased from 63.2% to 72.5% following subsequent ctDNA. Targets for genotype-matched therapy guided by subsequent ctDNA alone commonly included PARP, immune checkpoint, and BRAF. The ORR was 23.2% and 26.7% (*P* = .75), and the median PFS was 5.2 and. 3.7 months (HR, 1.5 [95% CI, 0.79 to 2.80]) for genotype-matched therapy based on initial versus subsequent ctDNA alone, respectively.

**CONCLUSION:**

Combining ctDNA with tissue analysis, followed by sequential ctDNA assessments, effectively enhances the identification of actionable biomarkers. This strategy facilitates clinically beneficial, genetically informed therapies, underscoring its significant value in precision oncology.

## BACKGROUND

Comprehensive genomic profiling using next-generation sequencing has enabled more personalized treatment options by guiding the selection of targeted therapies according to identified genomic biomarkers for various types of cancers.^1-4^ Although tumor tissue genotyping remains the standard practice, tumor tissue samples are sometimes unavailable or their use is inappropriate owing to poor quality or insufficient quantity, which results in sequencing failure.

**CONTEXT**

**Key Objective**
Using a nationwide cancer genome screening project (SCRUM-Japan MONSTAR-SCREEN-1), we evaluated the clinical utility of combined circulating tumor DNA (ctDNA) genotyping (combining ctDNA with tissue and longitudinal ctDNA genotyping) including the efficacy of genotype-matched therapies guided by their findings.
**Knowledge Generated**
Combining ctDNA with tissue genotyping and sequential ctDNA genotyping increased the detection of actionable biomarkers. The efficacy of genotype-matched therapies guided by these additional ctDNA results was comparable with that of baseline tissue and baseline ctDNA genotyping.
**Relevance**
Combining ctDNA with tissue genotyping and longitudinal ctDNA genotyping identifies additional patients who may benefit from genotype-matched therapy.

Recently, plasma-based circulating tumor DNA (ctDNA) genotyping has become more widely used in cancer care because of its minimal invasiveness and rapid turnaround time.^1,5-10^ Previously, we demonstrated that ctDNA genotyping significantly increases enrollment in genotype-matched clinical trials compared with tissue screening without compromising treatment efficacy in patients with advanced GI cancers.^1^ Therefore, the impact of ctDNA genotyping on genotype-matched targeted therapy warrants further investigation with regard to non-GI cancers.

Furthermore, ctDNA genotyping theoretically permits the assessment of spatial tumor heterogeneity across all tumor sites, an evaluation that is impossible with single-lesion biopsies only.^11-13^ In addition, the sequencing of ctDNA present in longitudinally collected blood samples can facilitate identifying any resistance-related alterations that may emerge during treatment.^6,11-17^ Using ctDNA genotyping to capture tumor heterogeneity has the potential to broaden the patient population that may benefit from genotype-matched targeted therapy by supplementing conventional tissue genotyping methods. Importantly, ctDNA analysis can also identify de novo resistance alterations that might preclude the use of certain therapies, thereby avoiding ineffective treatments and allowing for more appropriate therapeutic selection up front. However, large-scale profiling of ctDNA and tissues remains necessary.

SCRUM-Japan MONSTAR-SCREEN-1 is one of the largest cancer genome screening projects aimed at profiling genomic alterations in both ctDNA from longitudinally collected blood samples and tumor tissues of patients with advanced solid tumors.^18,19^ Based on the identified biomarkers, the patients received genotype-matched targeted therapies through either clinical practice or clinical trials, and clinical data from these enrolled patients were prospectively collected for quality assurance. Here, we aimed to evaluate the clinical utility of sequential ctDNA genotyping, including the efficacy of tissue-combined and longitudinal ctDNA genotyping-guided genotype-matched targeted therapies using data from more than 2,000 patients with advanced solid tumors in SCRUM-Japan MONSTAR-SCREEN-1.

## METHODS

### Study Design

**FIG 1.**
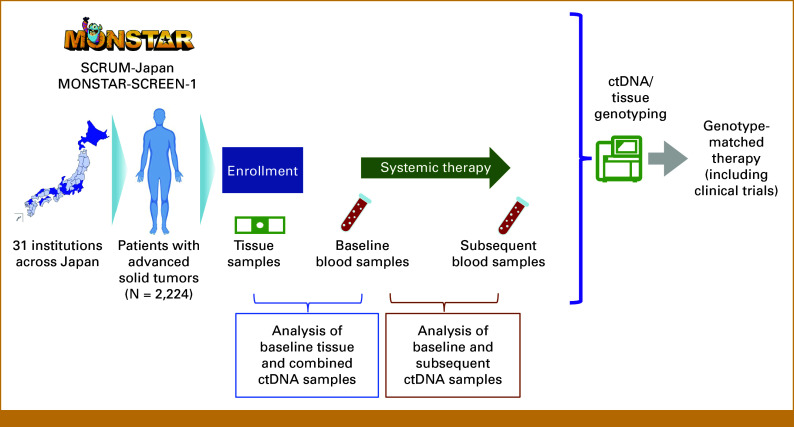
Study schema of the SCRUM-Japan-MONSTAR-SCREEN-1 study. ctDNA, circulating tumor DNA.

SCRUM-Japan MONSTAR-SCREEN-1 is a nationwide cancer genome screening project with the primary objective of prospectively profiling plasma and tumor tissues of patients with advanced solid tumors from 31 Japanese institutions (UMIN 000036749; Fig 1). Key eligibility criteria included the presence of histologically confirmed unresectable solid tumors, age ≥16 years, previous or planned systemic therapy, an Eastern Cooperative Oncology Group performance status of 0-1, and adequate organ function. Blood samples were collected twice from each patient, once before treatment initiation and again after disease progression, to monitor alterations in the ctDNA genomic profile that occurred during systemic therapy. For patients with previous palliative systemic therapy, blood samples were collected at the time when the disease progressed during the previous systemic therapy and before the start of the subsequent systemic therapy. Tumor tissue genotyping was performed in two scenarios: (1) patients who had not yet received any palliative systemic therapy for metastatic disease and (2) patients who had received previous therapy only in the curative-intent setting (neoadjuvant and/or adjuvant therapy was permitted). For patients who had already received palliative systemic therapy for metastatic disease, only blood-based testing was performed.

While tissue and blood underwent concurrent sequencing analysis, their collection times differed. Tissue samples were archival diagnostic specimens obtained before study enrollment, whereas blood samples were collected prospectively at study entry. This pragmatic approach reflects real-world clinical practice where molecular analysis of archival tissue and contemporary blood samples guides treatment decisions, rather than serving as a concordance study between simultaneously collected specimens.

The MONSTAR-SCREEN-1 study was conducted in accordance with the Declaration of Helsinki and the Japanese Ethical Guidelines for Medical and Health Research Involving Human Subjects. The study protocol was approved by the Institutional Review Board of each participating institution. All patients provided written informed consent before participating in this observational study. This study was initiated in July 2019, and enrollment was completed in February 2022.

### ctDNA and Tissue Genotyping

Blood samples and tumor tissues were profiled using FoundationOne Liquid CDx (F1LCDx; Foundation Medicine, Cambridge, MA) and FoundationOne CDx (F1CDx; Foundation Medicine), respectively. Genotyping was conducted in a Clinical Laboratory Improvement Amendment–certified, College of American Pathologist–accredited, New York State–approved laboratory (Foundation Medicine).

For ctDNA genotyping, circulating cell-free DNA was extracted from whole blood and analyzed using F1LCDx, a validated in vitro diagnostic device that targets 324 cancer-related genes. A complete list of these genes is shown in the Data Supplement. The assay uses hybrid capture technology and deep sequencing coverage to report single-nucleotide variants, indels, genomic rearrangements, copy number amplifications and losses, and genomic signatures, including blood tumor mutational burden (TMB), microsatellite instability (MSI), and ctDNA fraction.^20^

For tissue genotyping,^21^ the pathologic diagnosis of the tissue biopsy was confirmed using standard hematoxylin and eosin–stained slides, and all samples forwarded for DNA extraction contained a minimum of 20% tumor nuclei. Specifically, 50-1,000 ng of DNA was used for whole-genome shotgun library construction and hybrid capture of the same 324 cancer-related genes as those of F1LCDx and analyzed using F1CDx. The assay uses deep sequencing coverage to report single-nucleotide variants, indels, genomic rearrangements, copy number amplifications and losses, and genomic signatures, including TMB, MSI, and loss of heterozygosity.

### Therapeutic Selection and Clinical Data Collection

The actionability of genomic alterations was classified using the OncoKB knowledge base and represented the sufficiency of evidence for the gene as a predictor of drug sensitivity.^22^ We defined actionable alterations using OncoKB level 1-3 evidence to maintain consistency with established guidelines and previous studies. While this approach may identify alterations with varying levels of clinical evidence across different tumor types, it reflects current practice in molecular tumor boards and clinical decision making. Patients were generally treated according to the guidelines for their cancer type, with enrollment in clinical trials of genotype-matched targeted agents based on their genotyping results. The clinical trials included company-sponsored and investigator-initiated trials.

Clinicopathologic information and efficacy data for systemic therapy were collected using an electronic data capture system. All clinical data were periodically updated and finalized by combining auto-generated and manually added queries from the SCRUM-Japan Data Center.

### Statistical Analysis

Differences in proportions were assessed using the Fisher’s exact test and the chi-square test. Tumor response to systemic therapy was assessed according to RECIST version 1.1. Progression-free survival (PFS) was estimated as the time elapsed from the date of treatment initiation to the date of disease progression according to the investigator's assessment or the date of death from any cause. The Kaplan-Meier method was used to estimate survival rates, and treatment groups were compared using the log-rank test. Multivariate analysis of PFS according to detection status was conducted using the Cox proportional hazards model, with calculation of the hazard ratio and 95% CI. Sample size calculations were not performed because the present study was observational. Statistical analyses were performed using R v4.1.1 (R Project for Statistical Computing). The data cutoff date for the analyses was March 31, 2023. Statistical significance was set at *P* < .05. False discovery rate correction was used to control type I error.

## RESULTS

### Efficacy of Genotype-Matched Therapy Guided by ctDNA Genotyping and Tissue Genotyping

**FIG 2.**
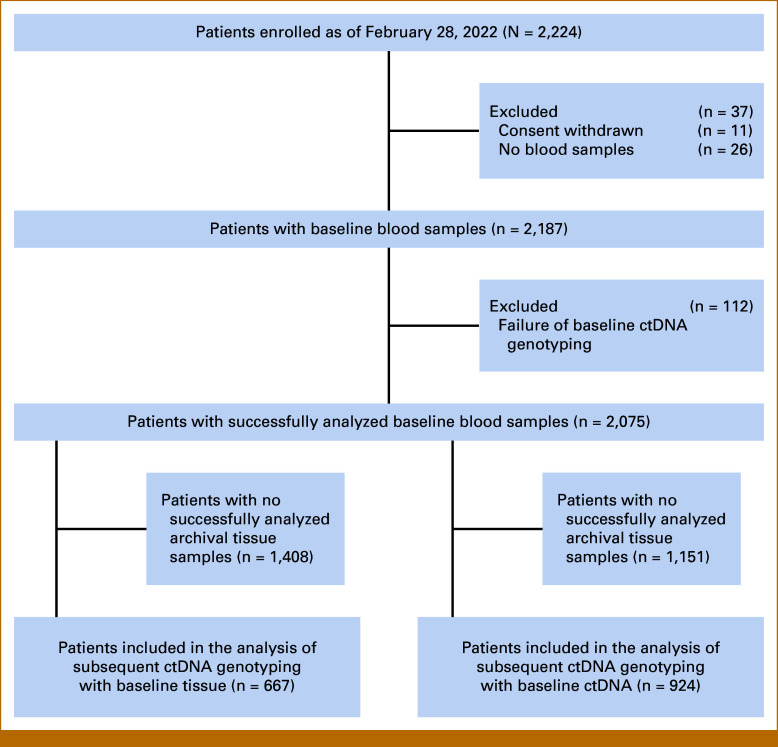
CONSORT diagram of enrolled patients from the SCRUM-Japan MONSTAR-SCREEN-1 study. ctDNA, circulating tumor DNA.

Of 2,224 patients enrolled in MONSTAR-SCREEN-1, 2,187 who underwent ctDNA genotyping were included (Fig 2). For the 667 patients who had both tissue and ctDNA genotyping results available, it is important to note that while sequencing analyses were performed concurrently, the actual sample collection times differed. Tissue samples were typically from archival diagnostic biopsies. The median interval between collection of tissue and blood samples was 1.5 months (range, 0.03-177.9). This approach reflects real-world clinical practice where contemporary blood samples are often analyzed alongside historical tissue specimens for treatment decision making. The baseline characteristics of the patients are summarized in the Data Supplement.

**FIG 3.**
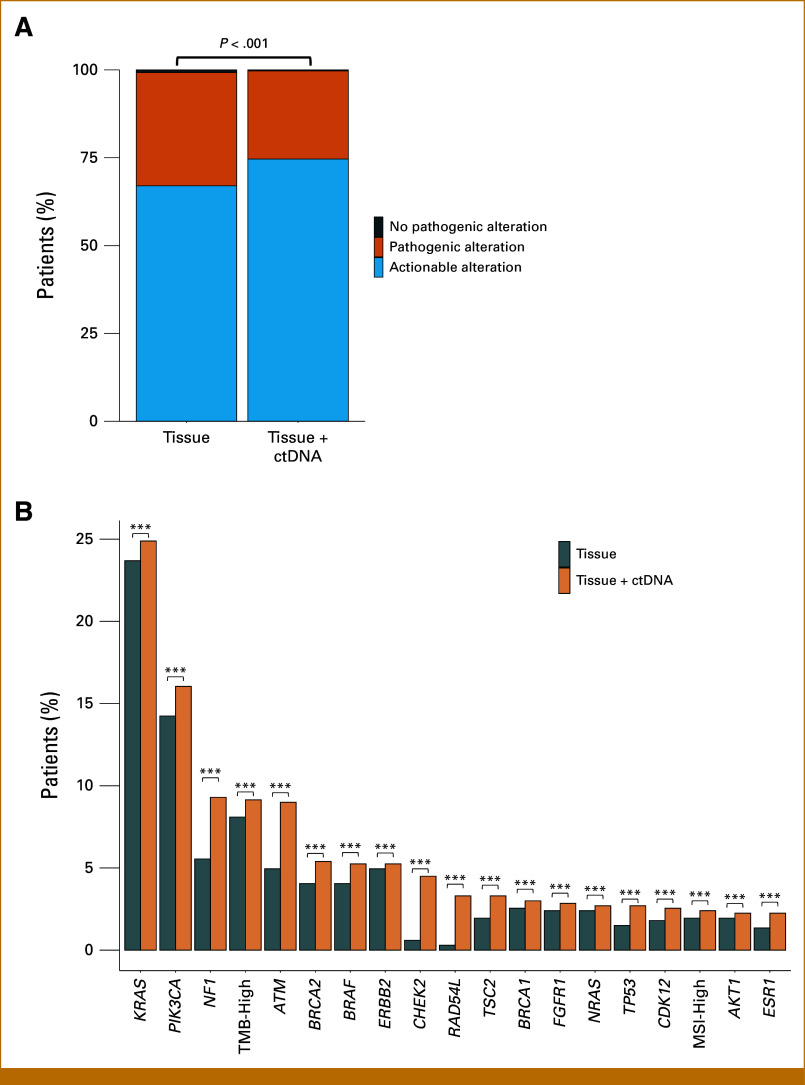
Detection of actionable alterations by subsequent ctDNA genotyping in patients with baseline tissue genotyping. (A) Percentage of patients with pathogenic or actionable alterations detected by baseline tissue genotyping or baseline tissue and ctDNA genotyping. (B) Proportion of actionable biomarkers detected with tissue genotyping alone or tissue combined with ctDNA genotyping. ***FDR—adjusted *P* < .005. ctDNA, circulating tumor DNA; FDR, false discovery rate; MSI, microsatellite instability; TMB, tumor mutational burden.

In 667 patients with both tissue and ctDNA genotyping results, the detection of actionable biomarkers significantly increased from 67.0% (447 of 667) with tissue genotyping alone to 74.7% (498 of 667) when combined with ctDNA genotyping (chi-square test, *P* < .001, Fig 3A). Similar significant increases were observed for specific actionable alterations as shown in Figure 3B.

**FIG 4.**
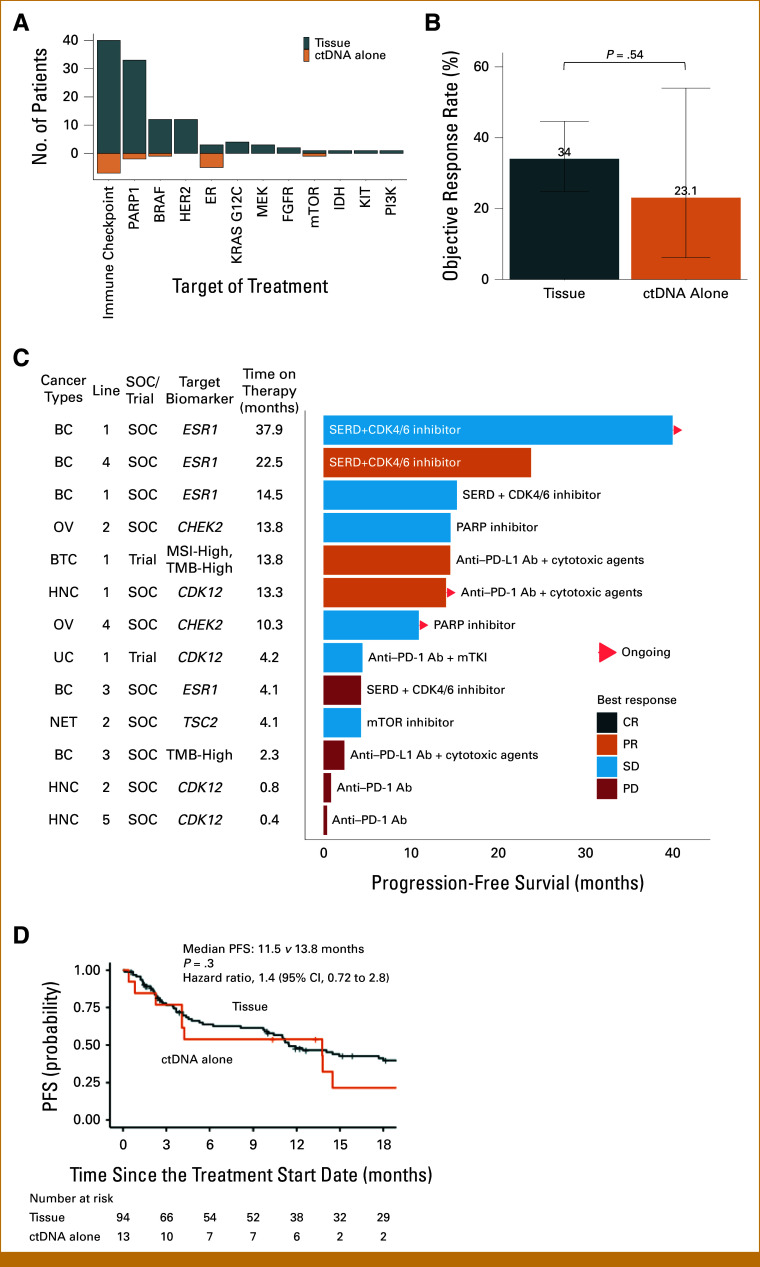
Clinical utility of subsequent ctDNA genotyping in patients with baseline tissue genotyping. (A) Treatment targets of genotype-matched therapy guided by baseline tissue or subsequent ctDNA genotyping. (B) Overall response rate of genotype-matched therapies guided by baseline tissue or subsequent ctDNA genotyping. Error bars represent 95% CIs. (C) Landscape of genotype-matched therapies guided by baseline tissue or subsequent ctDNA genotyping. (D) PFS of patients who received genotype-matched therapies guided by baseline tissue or subsequent ctDNA genotyping. Ab, antibody; BC, breast carcinoma; bTMB, blood tumor mutational burden; CR, complete response; CRC, colorectal carcinoma; ctDNA, circulating tumor DNA; ER, estrogen receptor; GC, gastric carcinoma; HNC, head and neck carcinoma; MEL, melanoma; OV, ovarian carcinoma; PARP, poly(ADP-ribose) polymerase; PD, progressive disease; PFS, progression-free survival; PR, partial response; SD, stable disease; SERD, selective estrogen receptor degrader; SOC, standard of care; UC, urothelial carcinoma.

Among 129 genotype-matched therapies in 111 patients, 16 (12.4%) were informed by heterogenous alterations detected only by ctDNA genotyping, frequently targeting immune checkpoint, estrogen receptor, and poly(ADP-ribose) polymerase (PARP; Fig 4A).

Among 107 treatments assessable for response, those guided by ctDNA genotyping alone showed an objective response rate (ORR) of 23.1% (3 of 13) compared with 34.0% (32 of 94) for tissue-guided therapy. While these rates were not significantly different (*P* = .54, Fig 4B), the small number of ctDNA-only guided treatments (n = 13) limits definitive conclusions about comparative efficacy.

While our initial pooled analysis showed comparable outcomes between tissue and ctDNA-guided therapies, further stratification reveals important differences across disease types and therapeutic classes. For instance, in breast cancer, *ESR1* mutation detection by ctDNA led to an ORR of 25% (n = 4) with SERD therapy, whereas in ovarian cancer, DNA damage repair gene mutation detection resulted in an ORR of 0% (n = 2) with PARP inhibitors.

The utility of tissue versus ctDNA-guided therapy appears to vary by context, with certain biomarker-disease-therapy combinations showing particular promise for ctDNA-based detection. However, the small sample sizes in many subgroups limit definitive conclusions about relative efficacy.

Specific cases, such as treatments targeting the *ESR1* mutation in breast cancer, MSI and TMB-high in biliary tract cancer, and *CDK12* mutation in head and neck cancer—detected exclusively through ctDNA but not in tissue samples—demonstrated partial responses (Fig 4C). PFS was also comparable between the two groups, with medians of 11.5 (95% CI, 9.8 to 23.8) and 13.8 (95% CI, 4.1 to not reached) months (hazard ratio [HR], 1.4 [95% CI, 0.72 to 2.8]; *P* = .30), respectively (Fig 4D). The details of ORR and PFS by subgroup and details of genotype-matched therapies guided by ctDNA genotyping alone are summarized in the Data Supplement.

### Efficacy of Genotype-Matched Therapy Guided by Longitudinal ctDNA Genotyping

**FIG 5.**
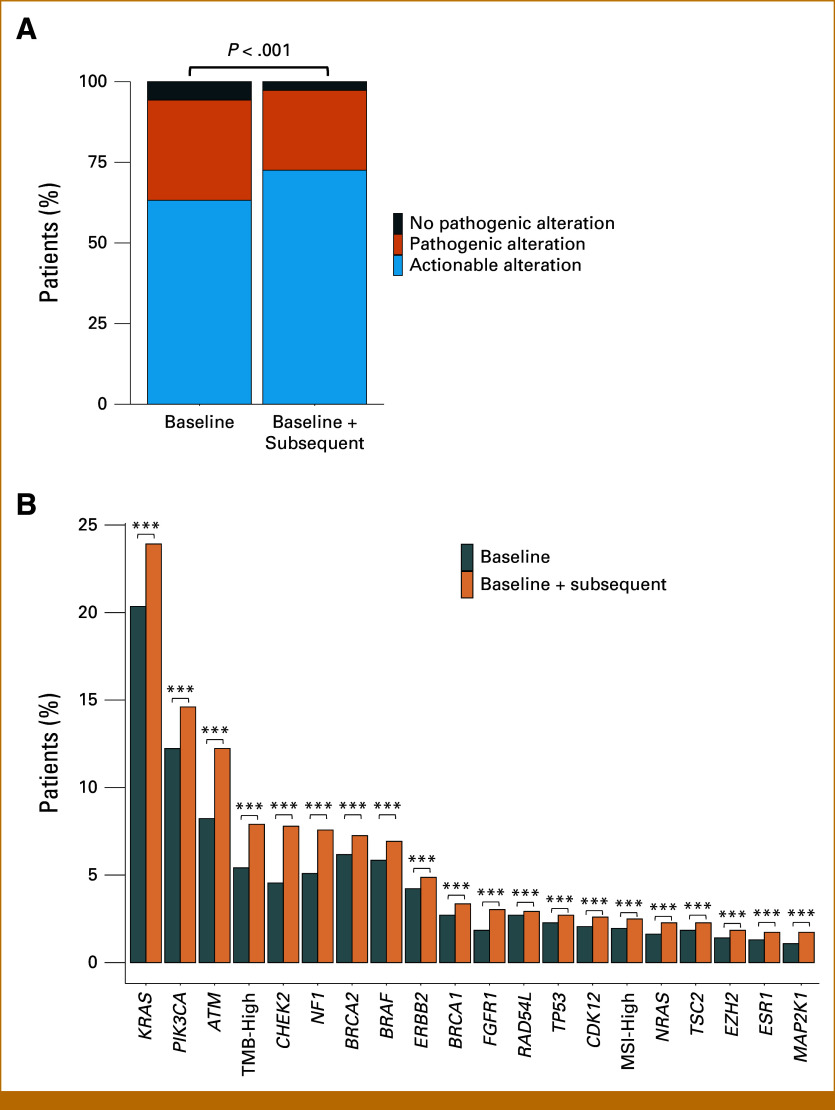
Detection of actionable alterations by subsequent ctDNA genotyping in patients with baseline ctDNA genotyping. (A) Percentage of patients with pathogenic or actionable alterations detected by baseline ctDNA genotyping only or baseline tissue and subsequent ctDNA genotyping. (B) Proportion of actionable biomarkers detected with baseline ctDNA genotyping alone or longitudinal ctDNA genotyping. ***FDR—adjusted *P* < .005. ctDNA, circulating tumor DNA; FDR, false discovery rate; MSI, microsatellite instability; TMB, tumor mutational burden.

Next, we analyzed efficacy of genotype-matched therapy based on longitudinal ctDNA genotyping. Both baseline and subsequent ctDNA genotyping results were available with 924 patients. The baseline characteristics of the patients are summarized in the Data Supplement. In 924 patients with both baseline and subsequent ctDNA results, actionable biomarkers were detected in 72.5% (670 of 924) of patients, which was higher than 63.2% (584 of 924) detection rate with baseline ctDNA alone (Figs 5A and 5B). To assess potential selection bias, we compared baseline ctDNA results between patients with single versus serial testing. The higher detection rate at baseline in patients who underwent serial testing (57.0% *v* 63.2%, *P* = .01) suggests some selection bias, with patients harboring actionable alterations being more likely to receive subsequent ctDNA testing. However, this modest difference does not fully explain the substantial increase in detection rate (to 72.5%) observed with serial testing, supporting the value of sequential monitoring in identifying newly emerging alterations.

**FIG 6.**
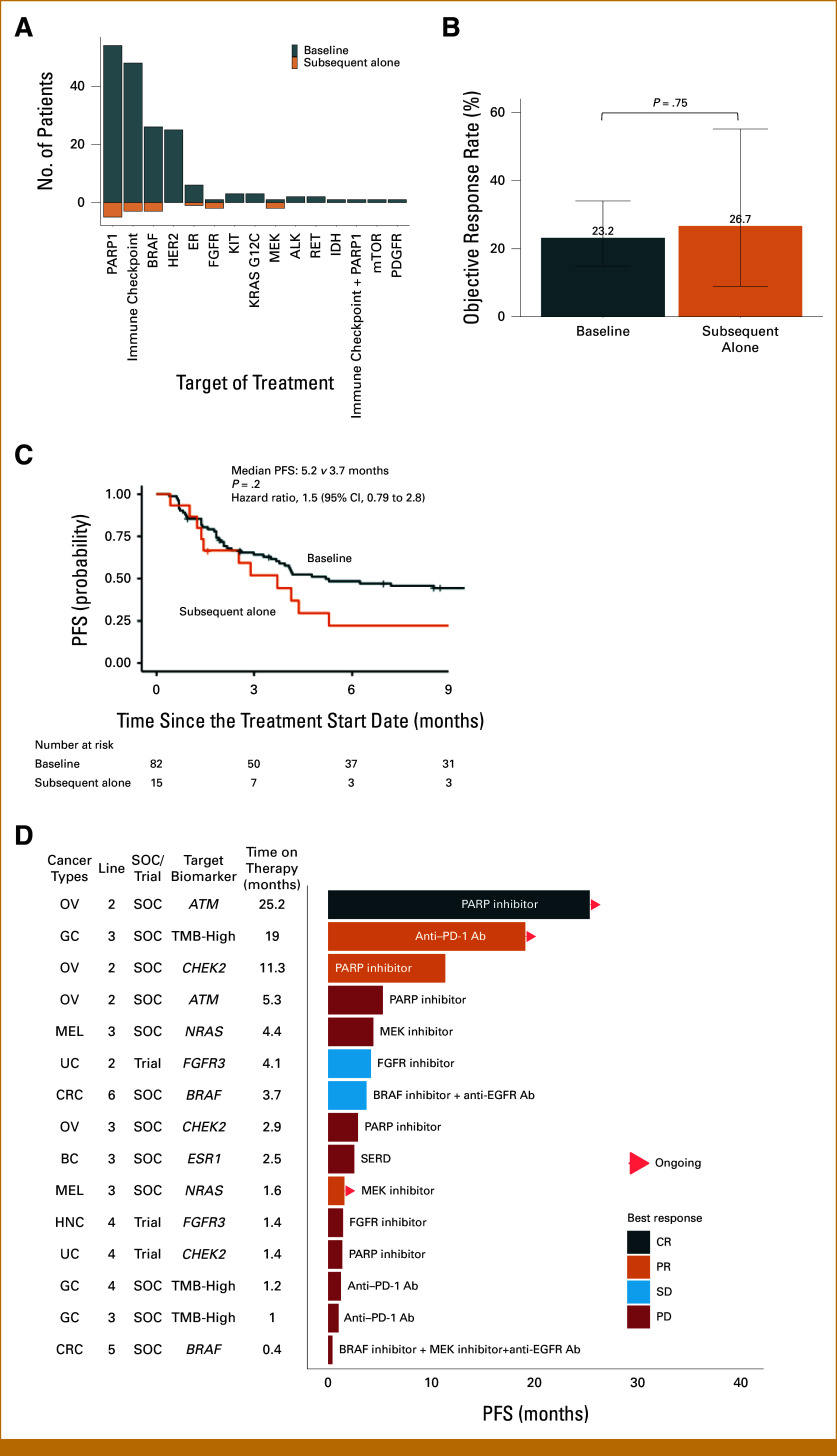
Clinical utility of subsequent ctDNA genotyping in patients with baseline ctDNA genotyping. (A) Treatment targets of genotype-matched therapy guided by baseline ctDNA or subsequent ctDNA genotyping. (B) Overall response rate of genotype-matched therapies guided by baseline or subsequent ctDNA genotyping. Error bars represent 95% CIs. (C) PFS of patients who received genotype-matched therapies guided by baseline or subsequent ctDNA genotyping. (D) Landscape of genotype-matched therapies guided by subsequent ctDNA genotyping only in patients with baseline ctDNA genotyping. Ab, antibody; BC, breast carcinoma; bTMB, blood tumor mutational burden; CR, complete response; CRC, colorectal carcinoma; ctDNA, circulating tumor DNA; ER, estrogen receptor; GC, gastric carcinoma; HNC, head and neck carcinoma; MEL, melanoma; mo, months; OV, ovarian carcinoma; PARP, poly(ADP-ribose) polymerase; PD, progressive disease; PFS, progression-free survival; PR, partial response; SD, stable disease; SERD, selective estrogen receptor degrader; SOC, standard of care; UC, urothelial carcinoma.

Of the 191 genotype-matched therapies in this cohort, 16 (8.4%) were guided by alterations detected only through sequential ctDNA genotyping, frequently targeting PARP, immune checkpoint, and BRAF (Fig 6A). To evaluate the clinical utility of sequential ctDNA genotyping, we compared the efficacy of therapies guided by alterations detected at baseline versus those detected only in subsequent ctDNA analysis. This comparison specifically addresses whether newly emerging alterations can identify therapeutic targets that provide clinical benefit comparable with those present at baseline. To ensure that the assessment of the efficacy was not biased by treatment line, analyses were focused on treatments initiated after enrollment, including 97 therapies in 87 patients. The ORR was similar between therapies informed by baseline versus subsequent ctDNA genotyping alone (23.2% *v* 26.7%, *P* = .75; Fig 6B). PFS was also not significantly different between both groups with the median PFS of 5.2 months (95% CI, 3.8 to 11.2) for treatments based on initial ctDNA and 3.7 months (95% CI, 1.5 to not reached) for those based on subsequent ctDNA findings (HR, 1.5 [95% CI, 0.79 to 2.8]; *P* = .20, Fig 6C). While the number of treatments based on subsequent ctDNA findings is limited, these initial findings suggest the potential value of sequential monitoring in identifying clinically relevant therapeutic targets. Notably, the detection of specific mutations exclusively through subsequent ctDNA results, such as *ATM* mutation in ovarian cancer, TMB-high in gastric cancer, *CHEK2* mutation in ovarian cancer, and *NRAS* mutation in melanoma, led to tumor responses, which exemplifies the potential of longitudinal ctDNA genotyping to uncover novel, actionable targets that may not be evident at the onset of treatment (Fig 6D). The details of ORR and PFS by subgroup and details of genotype-matched therapies guided by subsequent ctDNA genotyping are summarized in the Data Supplement.

## DISCUSSION

Using the SCRUM-Japan MONSTAR-SCREEN-1 network, we demonstrated that, within the same screening population and investigator network, ctDNA genotyping helped to identify additional patients who may benefit from genotype-matched therapy by detecting genomic alterations that were undetectable by tissue genotyping. In addition, subsequent ctDNA genotyping could detect novel genomic mutations that were not initially detected by baseline ctDNA genotyping, leading to identification of a clinically meaningful population receiving genotype-matched treatments. Our findings from examining this large cohort underscore the utility of ctDNA genotyping, not just as an alternative to tissue genotyping but also for identifying biologically actionable targets missed by tissue genotyping, expanding the population of patient populations suitable for precision oncology.

Combining ctDNA and tissue genotyping identified actionable biomarkers in a higher proportion of patients compared with tissue genotyping alone, consistent with previous studies that demonstrated the complementary value of ctDNA genotyping in detecting additional genomic alterations missed by tissue sampling alone.^6,13-15^ Notably, around 12% of genotype-matched therapies were informed by alterations exclusively detected through ctDNA genotyping, suggesting its complementary role in capturing tumor heterogeneity. Although we maintain our conclusion that ctDNA testing can identify potentially actionable alterations missed by tissue testing, we acknowledge that the small number of patients treated based on ctDNA-only findings (n = 13) represents an important limitation. The observed responses in this small subset provide preliminary evidence supporting the potential utility of ctDNA-guided therapy selection, but larger cohorts will be needed to definitively establish comparative efficacy.

While our study observed responses to therapies guided by both tissue and ctDNA-detected alterations, direct efficacy comparisons are limited by substantial heterogeneity in tumor types, alterations, and treatments. The absolute differences in response rates between tissue and ctDNA-guided therapies should be interpreted with caution given this heterogeneity and the relatively small number of ctDNA-only guided treatments. Rather than suggesting comparable efficacy, our findings demonstrate that ctDNA testing can identify potentially actionable alterations that might have been missed by tissue testing alone although the clinical benefit of acting on these findings requires further study in larger, more homogeneous cohorts.

The ability to detect emergent genomic alterations through longitudinal ctDNA genotyping is particularly valuable in the context of therapeutic resistance and clonal evolution. This finding aligns with previous studies on the utility of ctDNA in monitoring clonal evolution during treatment.^11^ By incorporating subsequent ctDNA analysis, an additional 9% of patients were identified with actionable biomarkers compared with baseline ctDNA genotyping alone. Importantly, the efficacy of genotype-matched therapies guided by these novel alterations detected through longitudinal ctDNA genotyping was similar to those based on baseline ctDNA findings, suggesting that these emergent alterations may represent clinically relevant treatment targets. This observation is consistent with previous reports highlighting the potential of ctDNA-guided therapy adaptation in overcoming acquired resistance.^16^

While our study demonstrates increased detection of actionable alterations through ctDNA genotyping, the clinical significance of these findings varies. Alterations in certain genes, such as *KRAS* mutations, showed modest but potentially more reliable increases in detection through ctDNA genotyping. These findings may better represent the true complementary value of concurrent plasma analysis as they are less likely to be confounded by clonal hematopoiesis (CH) and have well-established roles in guiding therapeutic decisions across multiple tumor types. This observation suggests that the utility of combined tissue and plasma testing might be best evaluated by focusing on such well-validated genomic alterations, rather than relying solely on the overall increase in actionable alterations.

The cases presented, such as responses to PARP inhibitors for *ATM* or *CHEK2* mutations and immune checkpoint inhibitors for blood TMB-high, exemplify the potential of longitudinal ctDNA genotyping to uncover actionable biomarkers that might have been missed by initial tissue or ctDNA analysis. These findings underscore the importance of continuous monitoring of tumor genomic landscape and the potential for ctDNA genotyping to guide treatment adaptation in the face of evolving tumor biology. Regarding responses observed to PARP inhibitors in patients with ovarian cancer with detected *ATM* and *CHEK2* alterations, we acknowledge several important caveats: (1) PARP inhibitors demonstrate activity in ovarian cancer independent of DNA damage repair gene mutations; (2) the detected alterations in DNA repair genes may represent CH rather than true somatic mutations; and (3) without matched germline testing or serial variant allele frequency monitoring, we cannot definitively distinguish tumor-derived from CH-derived alterations. Therefore, while responses were observed in these patients, we cannot conclude that the detected alterations were driving treatment benefit. Future studies incorporating matched germline testing and serial ctDNA monitoring with variant allele frequency tracking would be needed to better understand the biological significance of these findings in ovarian cancer.

Despite promising results, some limitations should be acknowledged. First, tissue genotyping remains an essential component of genomic profiling as not all alterations may be detectable in ctDNA, particularly in cases of low tumor burden or CH. In addition, the study did not directly compare the clinical outcomes of patients treated with genotype-matched therapies guided by ctDNA genotyping versus standard of care, limiting the ability to draw definitive conclusions about the impact on overall survival. Furthermore, the study focused on a specific ctDNA and tissue genotyping platform, and the generalizability of the findings to other platforms remains to be explored.

Several important limitations of our study must be acknowledged. First, the confounding effect of CH represents a significant challenge in interpreting ctDNA results, particularly for genes commonly affected by CH such as *ATM*, *CHEK2*, and other DNA repair genes. Without matched germline testing or specialized computational approaches, definitively distinguishing tumor-derived from CH-derived alterations remains difficult. This challenge is particularly relevant when evaluating serial samples as cytotoxic therapy may increase the prevalence of CH clones. Second, our broad definition of actionability, while inclusive, may overestimate the clinical utility of detected alterations. The relevance of specific alterations varies considerably by tumor type, and evidence supporting targeted therapy approaches differs substantially across cancer contexts. For instance, while *BRCA1*/*2* alterations have strong evidence supporting PARP inhibition in ovarian cancer, the clinical significance of other DNA repair gene alterations remains less clear in many tumor types. Finally, the application of these findings in clinical practice requires careful consideration of multiple factors including tumor type, treatment history, concurrent alterations, and the strength of evidence supporting specific therapeutic approaches. These limitations underscore the importance of expert interpretation of genomic findings within the specific clinical context.

It is essential to emphasize that the interpretation of genomic profiling results, whether from tissue or ctDNA, requires careful evaluation by experts who can integrate multiple factors: the specific tumor type, the patient's complete clinical context, detailed treatment history, and currently available therapeutic options. Molecular tumor boards, comprising multidisciplinary teams of oncologists, pathologists, molecular biologists, and genetic counselors, play a crucial role in this process. These experts can properly weigh the evidence supporting specific alterations as therapeutic targets, assess the likelihood that variants represent true somatic mutations versus CH, and consider how genomic findings fit within the broader context of standard treatment algorithms. This expert-driven approach is particularly important when evaluating novel or uncommon alterations, interpreting sequential genomic changes, and making decisions about experimental therapies or clinical trial enrollment.

In conclusion, the MONSTAR-SCREEN-1 study provides compelling evidence for the clinical utility of ctDNA genotyping, both in combination with tissue genotyping and through longitudinal monitoring, in guiding genotype-matched targeted therapies for patients with advanced solid tumors. The ability to capture tumor heterogeneity and evolving genomic landscapes through ctDNA genotyping may inform more personalized treatment strategies and potentially improve clinical outcomes.
